# Center of pressure limb path differences for the detection of lameness in dogs: a preliminary study

**DOI:** 10.1186/s12917-019-1881-1

**Published:** 2019-05-08

**Authors:** Sergio López, José M. Vilar, Mónica Rubio, Joaquin J. Sopena, Elena Damiá, Déborah Chicharro, Angelo Santana, José M. Carrillo

**Affiliations:** 1 Instituto Universitario de Investigaciones Biomédicas y Sanitarias, Universidad de las Palmas de Gran Canaria, Arucas, Las Palmas, Spain; 2 Departamento Medicina y Cirugía Animal, Cátedra García Cugat, Universidad Cardenal Herrera-CEU, CEU Universities, Valencia, Spain; 3 Departamento de Matemáticas, Universidad de las Palmas de Gran Canaria, Las Palmas, Spain; 4 Departamento de Patología Animal, Universidad de las Palmas de Gran Canaria, Arucas, Las Palmas, Spain

**Keywords:** Balance, Center of pressure, COP, Dog, Statokinesiogram

## Abstract

**Background:**

The limb center of pressure (COP) path measures and quantifies the load distribution within a limb in a still or moving subject. Under this premise, the aim of this study was to test whether data derived from this parameter could detect the differences between sound and lame limbs in unilaterally lame dogs with elbow dysplasia.

To accomplish this purpose, ten unilaterally lame dogs of similar conformation were walked over a pressure platform. Next, the COP path, in relation to the position of sound and lame limbs, was measured in a coordinate system over a standard paw template obtained by pedobarography during the whole support phase. To compare variables, force platform data (peak vertical force and vertical impulse) from the same animals were obtained. Sound and lame limb statokinesiograms were also obtained while the animals stood still.

**Results:**

The statistical analysis clearly showed that COP in lame limbs start cranially and were shorter than sound limbs. In addition, the value of the COP excursion index was lower in lame limbs. Finally, the area of statokinesiograms was greater in lame limbs.

**Conclusion:**

This methodology based in limb COP characteristics serves to discriminate between sound and lame limbs in dogs with elbow dysplasia.

**Electronic supplementary material:**

The online version of this article (10.1186/s12917-019-1881-1) contains supplementary material, which is available to authorized users.

## Background

Various methods to analyze the locomotor status within the veterinary field have been developed in order to generate useful parameters from both kinematic and/or kinetic perspectives. These methodologies should be able to provide accurate and reliable data and, if possible, form a set of parameters that will allow for the normal/abnormal static/dynamic events from a wide perspective. This invariably requires the use of more sophisticated systems [1].

These data should ultimately serve to detect lameness, and, among them, the center of pressure (COP) position may be considered the net output variable of interaction between all of the forces and torques that occur in the body (bCOP) or limb (lCOP) and its inertial properties. The COP position over time is named the COP path. This parameter quantifies the dynamic load distribution under the foot [2]. The lCOP path characteristics obtained in moving subjects provide insights into foot dynamics during the support phase of gait in human and, potentially, in animal species [3–6]. In this sense, it has been able to reliably detect biomechanical modifications due to neurological deficits, such as Parkinson’s [7], Hemiparesis [8] or even pain [3], in humans.

The main lCOP pathway characteristics that have been reported as useful are: 1) craniocaudal COP excursion (measured as an initial and final COP relative coordinates) [8]; 2) lateromedial displacement of the lCOP by means of the center of pressure excursion index (CPEI), which represents the lCOP path lateromedial excursion relative to limb width and multiplied by 100 to obtain this data in terms of percentage [3, 9].

The COP path can be also obtained in a standing position and records its resultant area during a determinate period of time. This parameter is named statokinesiogram, and its value shows body or limb balance [10].

In the veterinary field, previously published studies only examine the bCOP path [11–13]; more recently, the bCOP path’s efficacy for the detection of lameness in ponies at walk has been settled [14]. In dogs, bCOP modifications in unilaterally lame animals with elbow dysplasia (ED) have also been reported [15].

Regarding ED, this is a complex syndrome, where different factors could lead to a growth incongruence between the radius and ulna. Over time, ED causes joint damage, pain, and lameness [16, 17].

The hypothesis of this study was to prove that certain lCOP path characteristics are different in lame and sound limbs in dogs at walk and while standing still. For this reason, the aim of this study was to set a number of lCOP pathways –derived data that could serve to detect lameness in dogs with unilateral ED.

## Methods

### Animals

This study utilized 10 client-owned, adult dogs with similar conformation (2 rottweiler, 3 labrador retriever, 1 golden retriever, 2 german shepherd, 2 belgian shepherd). The body weight of the enrolled dogs ranged from 30 to 41,8 kg, and the ages were from 3 to 9 years.

Inclusion criteria comprised of the presence of weight-bearing unilateral forelimb lameness due to OA secondary to elbow dysplasia. The lameness of every dogs reached a score of 3–4 in a scale of 0–5 [18].

Furthermore, no medication could have been administered 1 month prior to the analysis.

To confirm or rule out OA, three standard radiographic views of both elbow joints (a lateral extension, lateral flexion, and a 15° oblique craniomedial caudolateral) [19] were taken under sedation with dexmedetomidine 10 ± 20 μg/kg (Dexdomitor, zoetis, Spain). Standard radiographs of stifle and hip joints were also taken in order to exclude other reasons for the observed clinical signs.

A complete clinical evaluation (physical examination, including vital signs and neurologic and orthopedic exams) assured that general health was otherwise normal.

### Pressure platform study

A Pressure platform (EPS/R1, Loran Engineering, Bologne, Italy) was used for this study. This device contains a total of 2096 pressure sensors of 1 cm2 distributed in an area of 48 × 48 cm. The range of pressure was set from 30 to 400 kPa.

The procedure for the dynamic and static pressure platform analysis has been previously published [15, 20]; briefly, dogs were leash guided by their owners over the pressure platform at a walk (velocity 1.2 ± 0.2 m/s; acceleration ± 0.2 m/s^2^). Velocity and acceleration were measured with a motion sensor (PS-2103A, Pasco®, California, USA) placed within the dogs trajectory. Three trials were recorded at a sampling frequency of 100 Hz from each dog. A trial was considered valid when the studied limb fully supported over the pressure platform and when the dog walked next to the owner without pulling on the leash and without head turns. The pressure platform was interfaced with a dedicated computer using Biomech® (Loran Engineering, Bologna, Italy) software. Once the images were isolated, the paws’ length was normalized to a fixed value of 9 cm, and width was then proportionally modified. Measurements were taken with a reference to an X-Y coordinate system.

Statokinesiograms were obtained while the dogs were placed in a quiet stance with their thoracic limbs over the pressure platform, perpendicular to the ground. The dog’s owner remained in front of the animal to attract the dog’s attention at a close distance. Three trials of 20-s recordings were obtained from each animal. A trial was considered valid when the animal remained with immobile limbs, tail and head during the whole 20 s recording procedure.

**Fig. 1:**
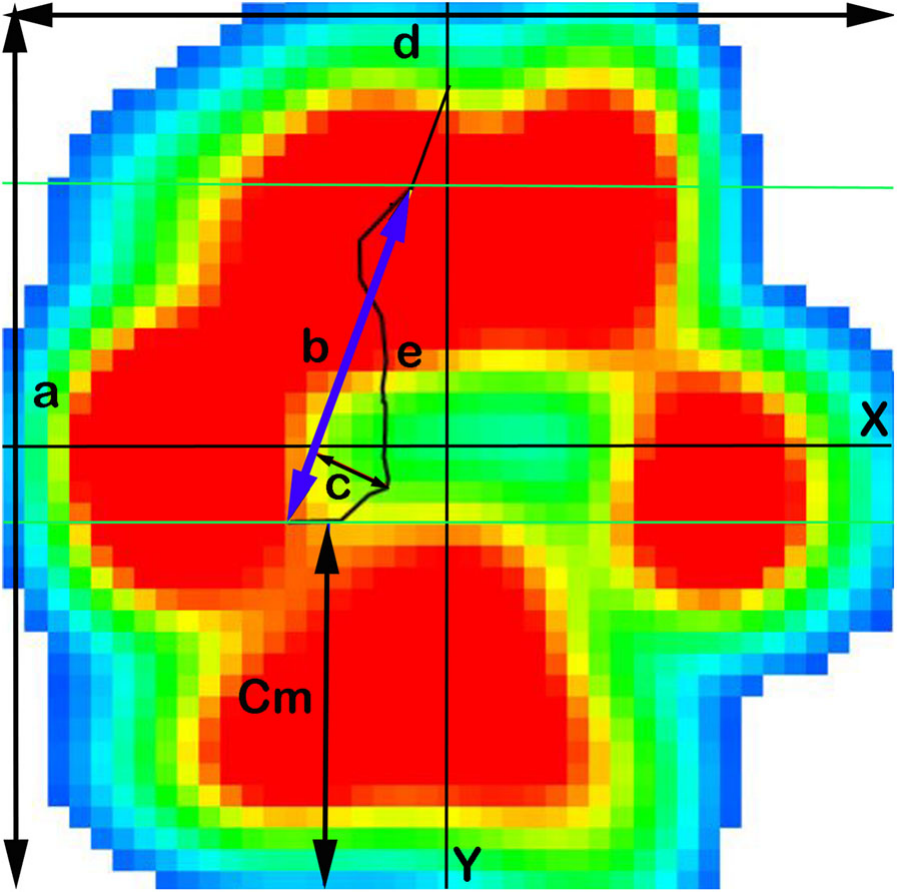
Paw podobarographic print with coordinate system and measurements made. X: X coordinate; Y: Coordinate; a: paw length; b: COP length; c: lCOP width; d: paw width; e: COP path length; Cm: caudal margin

The following were the obtained measurements (Fig. 1):

1. Caudal margin (Cm): defined as the distance between the most caudal limit of the paw print and the most caudal limit of the lCOP path.
2. lCOP pathway length (e): the length of the line that joins the recorded points of the lCOP trajectory. Measured in cm.
3. Craniocaudal index (CrCI): determines the COP length (b) related to the paw length (a). This is obtained with the following formula: % = (b / a) × 100. Expressed as a percentage.
4. Center of the pressure excursion index (CPEI): determines the lateromedial excursion of the COP (c) related to the paw width (d). The formula was the following: % = (c / d) × 100. Expressed as a percentage.

Higher values of all the above parameters are associated with better limb support [3, 8, 9].

5. statokinesiograms: defined as the area determined by an ellipse that contains 90% of the recorded points of the COP trajectory [10]. Measured in mm2, a lower value means more stability [15, 21].

### Force platform analysis

A force platform (Pasco, California, USA) was placed adjacent to the pressure platform in such a way that recordings from animals were performed in the same session. DataStudio software (Pasco, California, USA) was used to obtain PVF (N) values from three valid trials. Mean values were normalized to body weight (%BW).

### Statistical analysis

For the data analysis, a linear mixed effects model was considered: for each response variable (COP Length, CPEI, etc), the status of the limb (lame/sound) is a fixed effects factor, while the dog is a random effects factor.

The model is as follows:

$$ \mathrm{y}\_\mathrm{i}\mathrm{j}\mathrm{k}=\upmu \_\mathrm{i}+\mathrm{b}\_\mathrm{j}+\upvarepsilon \_\mathrm{i}\mathrm{j}\mathrm{k},\mathrm{i}=1,\dots, 2\kern0.36em \mathrm{j}=1,\dots, 10,\kern0.36em \mathrm{k}=1,\dots, 3 $$

$$ \mathrm{b}\_\mathrm{i}\approx \mathrm{N}\left(0,\upsigma \_\mathrm{b}\ \right)\kern0.72em \upvarepsilon \_\mathrm{i}\mathrm{jk}\approx \mathrm{N}\left(0,\upsigma \right) $$

where:

1. y_ijk is the k-th measure (k = 1,2,3) on the limb i (i = sound/lame) of the dog j (j = 1…10)
2. μ_i is the (fixed) effect of limb status i. This parameter represents the mean value of the variable in the sound (lame) limb.
3. b_j is the (random) effect of dog j. Values of b_j are supposed to be normally distributed with mean 0 and standard deviation σ_b, so σ_b is the variability in the response of the dogs.
4. ε_ijk is the residual in the measure ijk. This variable is assumed to be normally distributed with the mean 0 and standard deviation σ.

Statistical analysis was performed with ‘R’ statistical language and environment, version 3.3.2. (https://www.R-project.org/). For assessing the validity of the model, a Shapiro-Wilk test is applied to test the normality of the residuals, and a Levene test is used to test homoscedasticity.

## Results

Mean weight (± SD) was 37.08 ± 3.76 kg, and age was 5.80 ± 1.99 years. The mean (± SD) values and 95% CI of all obtained parameters are shown in Table 1. All data were normally distributed and homoscedastic (*p* ≥ 0.25 and *p* ≥ 0.12, respectively).

**Table 1:** Mean ± SD, 95% confidence interval and difference between LL and CLs for CM, Cop Path Length, CrCI, PVF, VI and statokinesiograms. ^a^ means significant difference

	LL	CL	Difference
Cm (%)	44.85 ± 5.12	31.13 ± 7.61	^a^13.72 ± 0.94
	40.86, 48.84	27.14, 35.12	11.83, 15.61
COP Path Length (%)	42.00 ± 4.94	55.68 ± 9.92	^a^13.69 ± 0.97
	37.05, 46.95	50.73, 60.63	11.74, 15.63
CrCI (%)	31.07 ± 4.49	44.01 ± 6.75	^a^12.94 ± 1.23
	28.08, 34.06	41.02, 47.01	10.47, 15.42
CPEI (%)	4.57 ± 1.65	9.30 ± 1.78	^a^4.73 ± 0.35
	3.65, 5.49	8.38, 10.22	4.02, 5.44
PVF (%)	32.72 ± 4.66	71.12 ± 3.57	^a^38.40 ± 0.78
	31.13, 34.31	69.53, 72.71	36.84, 39.96
VI (%)	13.49 ± 1.32	22.93 ± 1.58	^a^9.44 ± 0.24
	12.80, 14.18	22.24, 13.62	8.96, 9.92
Statokinesiogram (mm^2^)	16.18 ± 6.10	5.70 ± 3.43	^a^10.48 ± 0.75
	13.16, 19.19	2.68, 8.71	8.98, 11.98

*Cm* Caudal margin, *CrCI* Craniocaudal index, *CPEI* Center of pressure excursion index

**Fig. 2:**
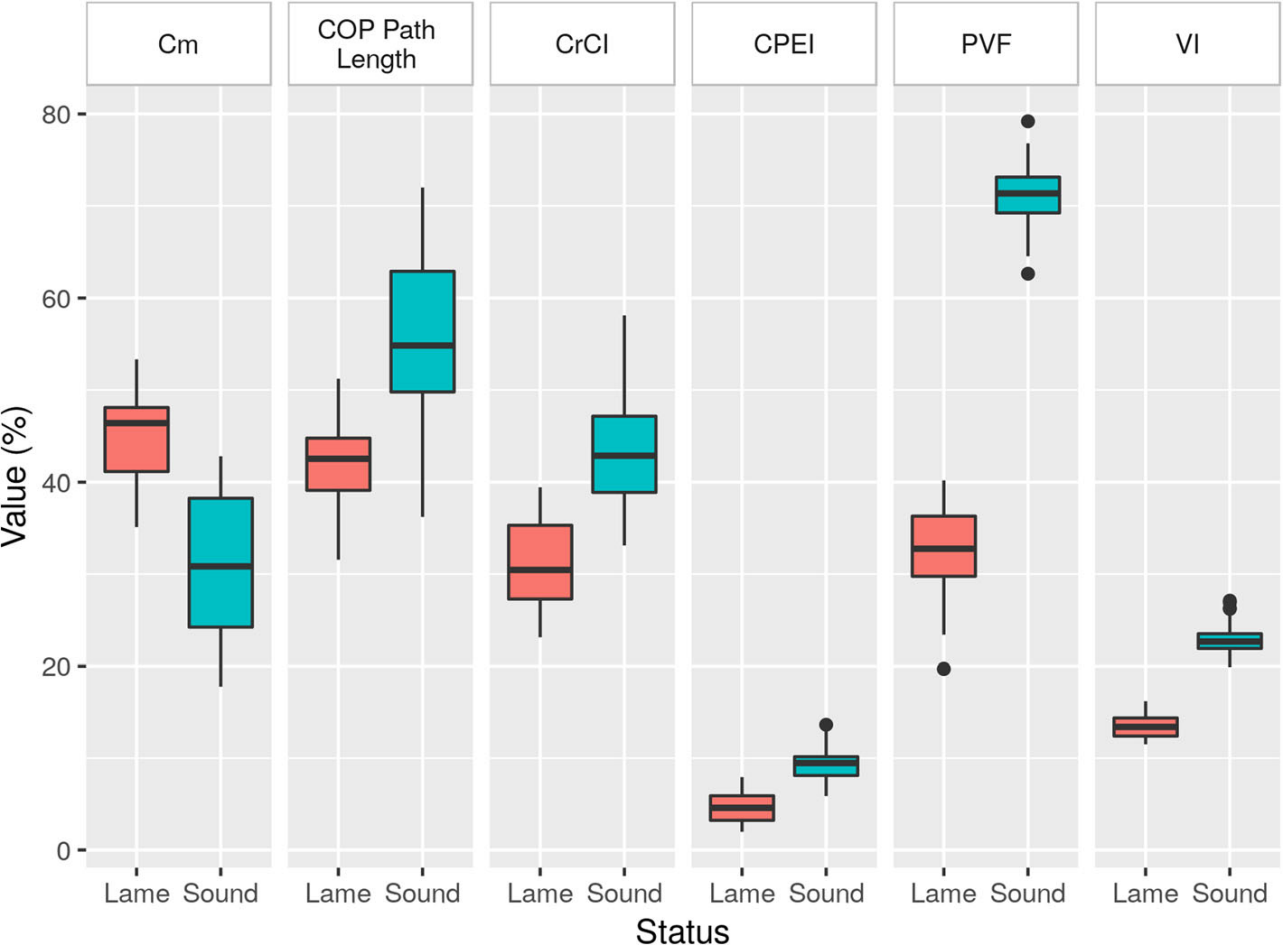
Boxplots showing differences in dynamic parameters between LL and CL. As can be seen, Cm values are lower in CL, while COP path Length, CrCI and CPEI indexes are higher when compared with LL. This also occurs in PVF and VI values


Significant differences between LL and CL were found in all cases (< 0.0001); concretely, a higher value of Cm and a lower COP Length, COP Path Length, and CrCI values in LL were observed when compared with CL. In the same manner, CPEI in LL were also lower than CL (Fig. 2, Additional file 1).

**Additional file 1:** **Video S1.** Limb and body statokinesiograms from a dog with a left limb lameness. As can be seen, the area of ellipse (18.28 mm^2^ Vs 8, 33mm^2^) in the left (red) LL is greater than the right (blue) CL. In the center (green) the body statokinesiogram can also be seen. (MP4 3152 kb)


In agreement with the data shown above, PVF and VI values also showed significant differences between LL and CL (*p* ≤ 0.0001) (Table 1). PVF and VI data were also normally distributed and homoscedastic (*p* ≥ 0.64 and *p* ≥ 0.51, respectively).

**Fig. 3:**
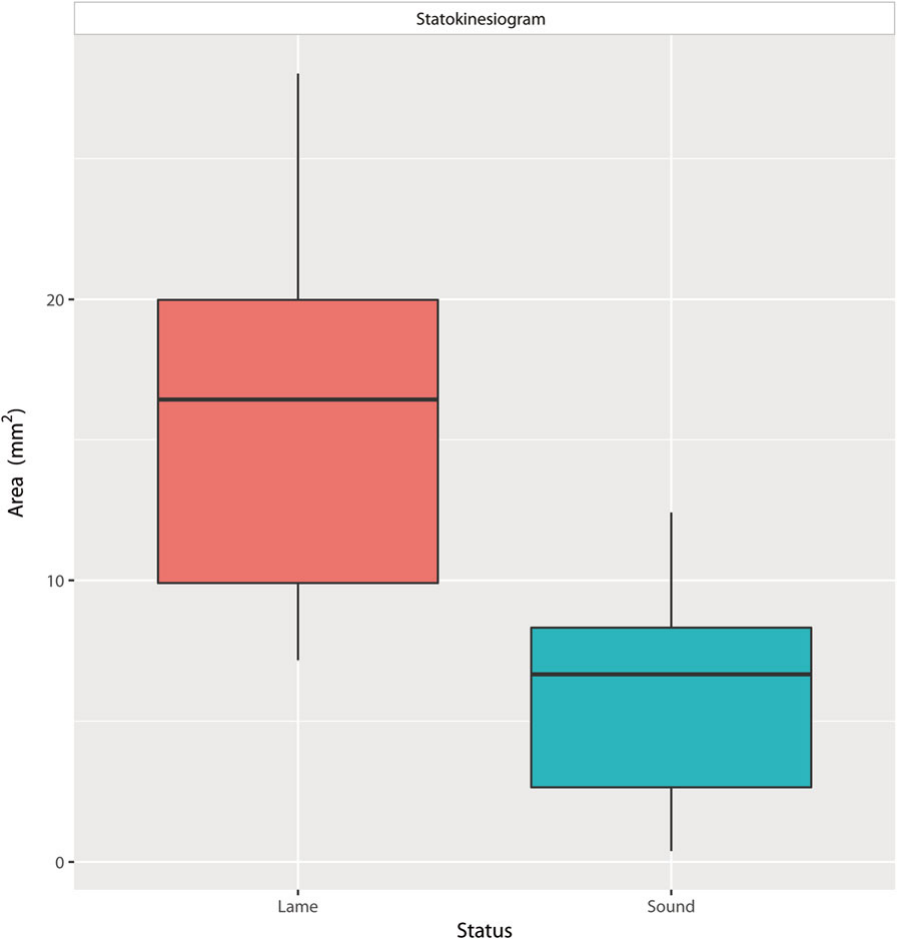
Boxplots of statokinesiogram (static) values of LL and CL. Area of LL is higher than LL i.e., more instable


Finally, the area from the statokinesiograms showed a higher value in LL (Fig. 3, Additional file 2). Additionally, a craniomedial COP slope was observed in both LL and CL when COP length was measured (Fig. 1, blue arrow).

**Additional file 2:** **Video S2.** Simultaneous videosequence of support phase in a CL (left) and LL (right). The lCOP (black point) path in LL starts more cranially and therefore shortened. (MP4 650 kb)


## Discussion

Our results provide a novel insight into the adaptive changes in lCOP characteristics in unilaterally lame dogs with ED.

To the best of our knowledge, no other previous studies exist regarding the clinical implications of dynamic and static lCOP path characteristics in lame dogs.

Limb weight load amount could be influenced by the gait speed or cadence and, consequently, could alter COP path patterns [22]. Acknowledging this possibility, we performed the study in a narrow range of velocity and acceleration and tried to enroll similarly sized animals in order to minimize severe cadence discrepancies.

Once the data were obtained, we assumed that measurements on caudocranial and mediolateral COP displacement would provide four basic differences between LL and CL regarding:

1. *The extent of net forward lCOP path progression*. Based in our results, lCOP path in LL is shortened and cranialized compared with CL. This is in concordance with other authors’ findings [8]. As made evident by the data, a larger Cm directly implies a shorter COP path length. This is invariably due to a shortened swing phase by a lack of limb extension, meaning the limb lands more vertically at the start of the braking phase [23]. This event prevents the metacarpal pad to exert a correct load absorption, expanding with the increase of weight-bearing when the limb lands [24, 25]. The impact shock could be, in the last instance, potentially transferred to muscles higher up the limb [5].
2. *Net mediolateral lCOP deviation*. As reported in previous research [26], a higher CPEI in CL is determined by an increased pad deformation, given that pad expansion is a direct response to weight loading. This effect has also been observed in human feet [9] and equine hooves [27].
3. *Statokinesiograms*. A greater area determines more instability [15]. This finding, although previously in reference to the body, remains true for limbs as well, since the area was greater in LL.
4. *The lCOP direction of progression in both sound and lame limbs*. As stated above, lCOP path described a certain angle (slope) as it pursued craniomedially with respect to the longitudinal axe of the paw. A possible explanation for this finding may be that the lCOP path follows the direction of the body’s center of mass and not the craniocaudal paw axe, which corresponds to other reports in humans [28].

Another interesting finding was that the lCOP caudocranial displacement is constant during the support phase, but velocity is not (Additional file 1), which coincides with reports in human research regarding sound limbs [8]. In the present study, this characteristic was evident not only in CL but also in LL.

In humans, longitudinal COP displacement corresponds to 83% of foot length and 18% of foot width [28]; their equivalent values in CL of our study with dogs were about 44% (CrCI) and 9% (CPEI), respectively, which is approximately half. Two facets could explain these differences: 1- that humans have plantigrad support, which starts in the calcaneus bone, whereas in dogs the support is digitigrade; 2- human bipedalism determines full load transfer to the support limb when walking, whereas dogs walk with two (or even three) limbs simultaneously sharing the load support.

The following are some limitations in our study:

1. The lCOP path patterns in sound limbs cannot be extrapolated to limbs from sound dogs. As in lame dogs, sound limb patterns are showing compensatory movements. For the same reason, data from unilaterally lame limbs should not be extrapolated to bilateral lameness.
2. Compensatory weight redistribution in lame dogs not only implies to the contralateral limb, as has been well established in dogs and horses [29, 30]; thus, it would be useful to obtain hind limb lCOP path values in a subsequent study. Moreover, it should be determined if any correlations exist between the lCOP path values with the lameness degree or lameness origin. Unfortunately, the relatively large dog sizes impede the simultaneous analysis of more than two limbs, and a larger platform pressure mat would be essential.
3. Parameters, such as Cm and CPEI, need to be qualitative and not quantitatively considered, given that cut-points were not defined in our study, although significant differences were found in our study between CL and LL. To establish an accurate limit value for soundness or lameness, a higher number of patients with the same characteristics (weight, conformation, or even breed) are necessary, as reported by others authors in similar human studies [4].
4. Finally, the number of lCOP characteristics assessed could represent a “signature” diagnosis of ED, where the kinetic parameters to detect it have been previously proven [23]. This also means that COP patterns in other musculoskeletal and neurodegenerative disorders could be quite different, which needs further investigation.

## Conclusion

This study showed that the lCOP path in LL is shorter, cranialized, and with smaller mediolateral excursion when compared with SL in dogs with unilateral ED. In addition, the lCOP path follows a craniomedial direction and not the paw longitudinal axe in both LL and CL. Its progression velocity is not constant.

## Abbreviations

bCOP: Body Center of Pressure
CL: Sound limb
Cm: Caudal margin
COP: Center of pressure
CPEI: Center of pressure excursion index
CrCI: Craniocaudal index
ED: Elbow dysplasia
lCOP: Limb Center of Pressure.
LL: Lame limb
PVF: Peak vertical force
VI: Vertical impulse
